# Effects of anti-parkinsonian medication on gait during internal and external rhythmic auditory cueing in PD

**DOI:** 10.3389/fnhum.2026.1808526

**Published:** 2026-04-22

**Authors:** Sidney T. Baudendistel, Lauren E. Tueth, Ryan P. Duncan, Allison M. Haussler, Liza Birov, Gammon M. Earhart

**Affiliations:** 1 Program in Physical Therapy, Washington University in St. Louis School of Medicine, Saint Louis, MO, United States; 2 Department of Neurology, Washington University in St. Louis School of Medicine, Saint Louis, MO, United States; 3 Department of Neuroscience, Washington University in St. Louis School of Medicine, Saint Louis, MO, United States

**Keywords:** gait, mental singing cues, musical cues, Parkinson’s disease, rhythmic auditory cueing

## Abstract

**Introduction:**

External and internal auditory cueing strategies improve gait function, including velocity and stride length, in people with Parkinson disease (PD). Further, internal cueing can reduce gait variability, a measure associated with falls. In the present study, we asked whether the efficacy of external and internal rhythmic cues depends on dopaminergic status, i.e., OFF vs. ON medication. This question is relevant to the real-world use of cueing, as dopaminergic medication responsiveness fluctuates for individuals. We measured gait while participants with PD walked with an external cue (music playing aloud) and with an internal cue (mentally singing) in both OFF and ON medication states. We hypothesized both cues would improve gait regardless of medication status.

**Methods:**

Participants (*n* = 36) walked at a preferred pace without cues (Uncued), while a song played aloud at 120% of each participant’s uncued cadence (Music), and while singing the same song in their head (Mental) in the OFF state and again after taking their normal dose of anti-PD medication. Variables were collected using Opal Sensors. Two, 2 (ON, OFF) × 3 (Uncued, Music, Mental) repeated-measures multivariate ANOVAs were conducted: one for mean gait outcomes (cadence, velocity, stride length, double limb support) and one for their linear variability.

**Results:**

Medication improved spatial measures of gait but did not change cadence. Both Music and Mental singing increased velocity, stride length, and cadence. For measures of linear variability, Music trials had statistically higher variability than Mental trials. There was an interaction effect between medication and cueing condition for double limb support; in the OFF-medication state, Music had longer double support times than Uncued and Mental singing.

**Conclusion:**

Cueing strategies improved gait regardless of medication status, suggesting they may be useful throughout the day despite medication fluctuations. However, internally-based mental singing cues produced greater benefits than external music cues, in both the ON and OFF state, for measures of gait variability. Further investigation of the efficacy of cues within and across anti-PD medication cycles would be helpful to understand their utility in the real world.

## Introduction

Parkinson disease (PD) is a progressive, neurodegenerative disease that impacts more than 11 million people globally (Su et al., 2025). This number is expected to rise to greater than 25 million by 2050, indicating a current and future challenge for public health worldwide (Su et al., 2025). For individuals with PD, slowness and instability during walking occur daily, limiting community engagement and reducing quality of life (Elbers et al., 2013; Santos García et al., 2019). Objective walking measures are associated with critical aspects of the disease. For example, short, shuffling steps, a key subjective observation of gait impairment, can be objectively measured with spatial measures of gait, as stride length decreases as the disease progresses (Wilson et al., 2020). Worsening variability including stride-to-stride linear variability of stride length and stride time are also related to disease progression (Wilson et al., 2020) and are often correlated to fall risk in healthy older adults and individuals with PD (Schaafsma et al., 2003; Hausdorff, 2009; Morrison et al., 2021). As objective walking measures can be collected non-invasively, there has been a special interest in integration of gait data into the clinical setting (Brzenczek et al., 2024).

The gold standard treatment for PD, carbidopa-levodopa, provides acute benefits to certain aspects of gait upon uptake of the drug. For instance, multiple studies confirm spatial measures of stride length significantly increase during straight ahead walking during the ON state compared to the OFF state, i.e., when participants have withdrawn from their medication overnight (Blin et al., 1991; Curtze et al., 2015; Smulders et al., 2016; Shah et al., 2025). However, temporal measures, such as cadence and stride duration, show mixed results (Blin et al., 1991; Almeida et al., 2007; Rochester et al., 2011; Curtze et al., 2015; Barbosa et al., 2024). Similarly, changes in gait variability, including stride time variability, also show little to no improvement with medication (Blin et al., 1991; Almeida et al., 2007; Rochester et al., 2011; Smulders et al., 2016; Su et al., 2023; Barbosa et al., 2024). Further, double limb support (DLS), a measure of dynamic stability (Mirelman et al., 2019), does not change even as speed increases when ON medication (Almeida et al., 2007; Curtze et al., 2015). In reality, the effects of medication ebb and flow throughout the day, as 52% of people with PD experience “wearing off” symptoms within the first year of medication usage (Stacy et al., 2005). After 5 years of medication usage, this number may increase to 100% of individuals (Stacy et al., 2005). The effects of levodopa on gait over the course of the levodopa cycle are highly variable between individuals and across days (MacKay-Lyons, 1998; Barbosa et al., 2024; Promsri and Federolf, 2025), necessitating the use of complementary strategies, such as cueing, to improve gait (Nonnekes et al., 2019).

External cueing, including rhythmic auditory cueing (RAC), remains highly effective in improving gait parameters as determined by multiple reviews (Lim et al., 2005; Spaulding et al., 2013; Rocha et al., 2014; Muthukrishnan et al., 2019; Forte et al., 2021; Harrison and Earhart, 2023; Burrai et al., 2024). These reviews demonstrate that RAC improves velocity and stride length (Agarwal et al., 2022; Burrai et al., 2024; Giorgi et al., 2024), but has mixed results with regard to gait variability (Harrison and Earhart, 2023). The overwhelming majority of investigations examining the effects of cueing are completed in the ON medication state (Harrison and Earhart, 2023; Burrai et al., 2024; Giorgi et al., 2024), as this state represents daily life. To our knowledge, only five studies have investigated the combined effects of anti-parkinsonian medication and RAC for individuals with PD (McIntosh et al., 1997; Almeida et al., 2007; Rochester et al., 2011; Rinaldi et al., 2014; Erra et al., 2019). Of those, only two statistically evaluated the interaction between medication and cue (Rochester et al., 2011; Erra et al., 2019). Rochester et al. (2011) investigated the effects of anti-PD medication on gait when using two different types of cues, via metronome and an attentional cue to “take big steps.” The authors reported that walking to a metronome set to 100% of uncued cadence reduced linear stride time variability compared to the uncued condition. Medication alone also reduced stride time variability, but the use of the metronome was more effective at reducing stride time variability than medication. More recently, Erra et al. (2019) investigated the use of RAC ON and OFF medication at three tempos, 90%, 100%, and 110% of baseline cadence. In general, medication improved gait but did not alter most measures of variability, as only swing time variability was reduced ON medication (Erra et al., 2019). RAC improved similar measures, including reducing swing time variability. There was no interaction between medication state and tempo of RAC, indicating that RAC at different tempos worked similarly regardless of medication state. Collectively, these studies demonstrate that external RAC improves gait regardless of medication state, but that medication status may uniquely influence the effect of RAC on temporal variability.

One of the main limitations of implementing RAC in a real-world setting is that a metronome or auditory cue is required to be played aloud. In a 2022 study, 87% of individuals with PD reported using a gait compensation technique in daily life, with the most popular being internal cueing strategies (Tosserams et al., 2022). Perhaps this is because internal cues may be employed covertly and without additional equipment. Our previous work established that mentally singing a song is a feasible form of internal RAC (Harrison et al., 2017, 2018). Specifically, mentally singing while walking increases stride length and velocity above uncued walking and to a similar level as walking with externally played music (Harrison et al., 2025). Further, mental singing reduces linear gait variability, while external music may have negligible effects or actually increase variability compared to uncued walking (Harrison et al., 2019, 2025). This increase in variability during external curing may be due in part to the need to synchronize gait to the external source of music, which requires consistent sensory integration and adjustment. Mental singing allows participants to create and maintain a song, potentially relying on other aspects of memory and motor planning networks (Halpern, 2001; Li et al., 2020) without external feedback. By removing the external cue, mental singing may allow for greater internal focus of the rhythmic cue, resulting in greater benefits while walking.

However, not all internally generated cues reduce variability. As mentioned, Rochester et al. (2011) tested the effect of an internal attention-based amplitude cue, asking participants to focus on “taking big steps.” This attentional, amplitude cue increased stride time compared to uncued walking in the ON state, but it did not alter stride time in the OFF state. Stride time variability and DLS variability also increased with the use of the attentional cue ON medication compared to the external RAC. The interaction effect between cue type and medication may be due to the difference in inherent demands of the cueing tasks and the selected outcome measures. External RAC targets the rhythm domain to increase speed of movement through reduced stride time. Conversely, the attentional cue to “take big steps” is a cue to increase amplitude of movement. As the basal ganglia are more involved with the scaling of movement than with the timing of movement (Blin et al., 1991; Morris et al., 1998), it is possible that the amplitude-based cue overcorrects amplitude when amplitude is already enhanced by medication.

Beyond potential overcorrection, anti-PD medication may impair important aspects of cognition and attention that could alter the ability to walk appropriately to a cue. There is evidence to suggest that anti-PD medication reduces cognitive inhibition (Roy et al., 2018), may impair motor sequence learning (Kwak et al., 2010; Ruitenberg et al., 2016), and reduces the ability to synchronize to an external cue (Almeida et al., 2007). The inconsistent findings regarding effects of anti-PD medication on gait and its potential role in cognition warrant further exploration of the effect that anti-PD medication, including levodopa, has on cued walking. Furthermore, previous research has not investigated the effect of anti-PD medication when using an internal RAC cue. The purpose of this study was to investigate the effects of anti-PD medication on walking among people with PD using external music cues and internal mental singing cues. We hypothesized anti-PD medication would improve spatial measures of gait in the uncued, preferred walking condition. Additionally, we hypothesized that external and internal rhythmic cues would improve both spatial (stride length) and temporal (cadence) measures of gait, regardless of medication status. In keeping with the results of our past studies (Harrison et al., 2019, 2025), including research suggesting that motor imagery is not different between medication states (Peterson et al., 2012), we hypothesized that mental singing would reduce gait variability more than external music, regardless of medication status.

## Methods

### Participants

Individuals were recruited as part of a cross-sectional study focused on investigating changes in motor ability, especially activities of daily living, in people with PD in the OFF state compared to the ON state of their normal anti-PD medication. The inclusion criteria were individuals aged 18 years and older, a diagnosis of idiopathic PD, and a stable medication regimen for at least 1 month prior to study entry. Self-reported tremor in dominant hand was a requirement of the larger parent study where a writing task was also utilized, although not included herein. The Telephone Montreal Cognitive Blind Assessment (T-MoCA) (Katz et al., 2021) was performed to ensure no significant cognitive impairment. Individuals who scored less than 18 out of a possible 22 on the T**-**MoCA were excluded. Other exclusion criteria included diagnosis of any other neurological condition or any unstable medical or concomitant illnesses or psychiatric conditions which, in the opinion of the investigators, would preclude successful participation. Individuals with deep brain stimulation were also excluded. Participation criteria, including the T-MoCA, and the informed consent were reviewed on the phone at least 1 day prior to the study visit to ensure participants were taking all medication as normal during the consent process.

All testing was completed within a single visit. Participants arrived in their OFF-medication state, defined as a minimum 12-h withdrawal of all PD-related medication. Participants completed the Movement Disorders Society – Unified Parkinson Disease Rating Scale, part 3 (MDS-UPDRS-III) followed by a series of walking tasks, as explained below. After the completion of all the walking tasks, participants were instructed to take their normal dose of medication. After a minimum of 1 hour, participants were asked if they felt like they achieved their “normal” medicated state. As optimal medication state is subjective, emphasis was placed on “normal” for the individual. If participants did not respond in the affirmative, participants were asked to let staff know when they were ready so that additional time could be provided if needed to reach full medication effect. As a majority of individuals are expected to feel like they are in the “ON-state” after 1 hour (Araújo-Silva et al., 2022), we anticipated 1 hour to be enough time for participants. MDS-UPDRS-III and the walking tasks were then completed again in the ON medication state.

### MDS-UPDRS-III & Hoehn and Yahr

The MDS-UPDRS-III is the gold-standard measure of motor symptom severity in individuals with PD with high between- and within-subject reliability and high construct validity (Goetz et al., 2008; Martinez-Martin et al., 2013; Evers et al., 2019). The assessment consists of 33 items, scored on a scale of 0 (no impairment) to 4 (maximum impairment). Scores on the MDS-UPDRS-III range from 0 to 132, with 132 indicating the worst possible severity of motor symptoms. The Hoehn and Yahr scale, which consists of a 1–5 scale focused on level of function, was also complete (Goetz et al., 2004). The Hoehn and Yahr scale has excellent Interrater and test–retest validity (Martinez-Martin et al., 2018).

The MDS-UPDRS-III & Hoehn and Yahr scale was performed by a certified rater and was video recorded. The rigidity items were scored by the in-person rater, and later, a separate team member rated the remaining items via blinded video review. This certified rater was blinded to medication status.

### Walking tasks

Three walking conditions were performed in both the OFF and ON medication states. First, participants completed a baseline walking condition (Uncued), in which they walked at their preferred, comfortable pace for 30 s with six Opal V2R sensors [APDM Wearable Technologies a Clario company, Portland, OR; (Mancini et al., 2011)] attached at the wrists, feet, sternum, and lumbar spine. MobilityLab (version 2) synchronizes these six sensors wirelessly, providing rapid data analysis with good to excellent validity for multiple measures of gait (Morris et al., 2019). Participants walked up and down a 100 ft. (~30 m) hallway. Three trials were completed to calculate the average Uncued cadence for each participant. The cadence calculated via MobilityLab was averaged across the three trials, multiplied by 1.2, then rounded to the nearest multiple of 5. This value, representing 120% of uncued cadence, was the cue tempo for the Music and Mental conditions. We previously established that cueing at 120% of baseline cadence exhibited the greatest improvements in stride length for greater than 80% of participants as compared to slower cadences (Harrison et al., 2025). All participants used an instrumental piano version of “Skip to My Lou”, a popular and enduring American folk song with simple lyrics. This song was pre-recorded and adjusted to 21 different tempos, 60–160 bpm at five beat increments. The open source audio editing software, Audacity (The Audacity Team, audacity.sourceforge.net/), was utilized to ensure key consistency between songs. Researchers confirmed that all participants were familiar with the song and could sing the song aloud while seated. External speakers played the song and all participants confirmed that the song could be heard well at all points in the hallway. Cued conditions were randomized.

For the Music trials, participants were instructed to “walk to the beat of the song”. Participants were asked to stand still and listen to the song at their personalized tempo for one round of the chorus. After this chorus, an auditory tone sounded, signaling the participant to start walking to the beat. The music looped continuously for 30 s until a second tone sounded to signal the end of the trial. This was completed three times.

For the Mental trials, participants were instructed to “sing the song in your head and walk to the beat.” Again, before every trial, participants stood still and listened to the song at their personalized tempo for one round of the chorus. When the beginning auditory signal sounded, the music was turned off, and the participant began walking to the song they sang in their head. After 30 s, the second tone sounded to signal the participant to stop. This was completed a total of three times.

Participants were asked if they needed a break after every trial and were allowed to rest whenever needed. Within cued conditions, short standing breaks of ~10 s were completed between the three trials. If longer breaks or seated breaks were requested, these were accommodated. Between cued conditions, longer, seated breaks were encouraged. At a minimum, standing breaks of 1 to 2 minutes were completed between conditions (Uncued, Music, Mental) to ensure data was collected appropriately as well as to review instructions for the next condition.

### Data analysis

MDS-UPDRS-III total scores and Hoehn and Yahr scale score were calculated for both OFF and ON medication states. To better describe the sample, tremor and postural instability and gait difficulty (PIGD) subscores were also calculated, similar to Stebbins et al. (2013). Tremor subscore included the sum of all tremor items (items 15–18). PIGD subscore was the sum of the gait, freezing of gait, and postural stability items (items 10–12).

Levodopa Equivalent Daily Dosage (LEDD), which allows for comparison between PD medication regimens, was calculated for each individual (Jost et al., 2023).

Gait variables included cadence, velocity, stride length, and DLS. Amplitude of stride-to-stride variability, calculated as coefficient of variation (CV), were also determined for each of these variables. Linear variability was chosen as it is more commonly used in the literature than non-linear variability, such as autocorrelations or fractal scaling (Harrison and Earhart, 2023).

### Statistics

Sample size was calculated for the parent study based on expected changes between OFF and ON medication status for the tremor items of the MDS-UPDRS-III. The number of participants needed for the study with 90% power and significance level *α* = 0.05 (two-sided) was 28 based upon an effect size of 0.61. However, it was expected some participants (~10%–15%) would not have substantial change in symptom severity when comparing OFF versus ON medication status (Pitz et al., 2020; Martin et al., 2021). Thus, we planned to recruit 36 individuals with PD to account for potential issues with lack of change in symptoms and technical issues. The sample size of 36 was expected to be more than adequate for the gait analyses, as prior studies in our lab with as few as 23 people have demonstrated significant effects of cueing on gait (Harrison et al., 2017).

Normality was evaluated through Kolmogorov–Smirnov tests and visual checking of histograms, box plots, and Q-Q plots.

MDS-UPDRS-III total score, tremor score, and PIGD score were tested using paired *t*-tests and, if not normal, Wilcoxon Signed-Rank tests (*p* < 0.05).

Due to the inherent relationship of gait variables, two separate 2 (ON, OFF) × 3 (Uncued, Music, Mental) repeated-measure multivariate analyses of variance (MANOVA) were implemented to control for significant correlations among variables. One MANOVA included the mean values of cadence, velocity, stride length, and DLS. The other analysis included the variability of those measures calculated as CV. Wilks’ *Λ* is reported for multivariate testing. Mauchly’s test was used to evaluate Sphericity and when significant, Greenhouse–Geisser corrections are reported. Partial eta squared (η_p_^2^) was also included [small (η_p_^2^ = 0.01 to 0.059), medium (η_p_^2^ = 0.06 to 0.139), and large (η_p_^2^ = 0.14 and above) effects (Field, 2013)]. Follow-up univariate analyses and *post-hoc* testing were completed when indicated. Marginal means with 95% confidence intervals and pairwise comparisons with 95% confidence intervals for the differences can be found within the supplement. Significance values of *p* < 0.05 with Bonferroni correction are reported.

## Results

### Data missingness and normality

There was no missing data among the individuals. For participant demographics, tremor subscore, PIGD subscore, and Hoehn and Yahr scale were not normally distributed due to their ordinal nature. The gait variables within the first MANOVA (cadence, velocity, stride length, and double limb support) were all considered normal. The variability variables within the second MANOVA (cadence CV, velocity CV, stride length CV, and DLS CV) were all considered not normally distributed due to skewness. All measures of variability were log-transformed similar to Lord et al. (2013). The statistics reported below are based on these transformed values. For reader clarity, untransformed averages of each variable are represented in the table and the figures.

### Participant demographics

Demographics of all 36 individuals are listed in Table 1. All individuals included were able to complete the 12-h withdrawal with an average duration of 1,029 min (17.15 h) since the last dose (Table 2). MDS-UPDRS-III total score, tremor subscore, and PIGD subscore all significantly improved after medication was taken (*p* ≤ 0.001, Table 2). Specifically, changes in MDS-UPDRS-III total score exceeded the what is considered minimal clinically important difference (Horváth et al., 2015). Hoehn and Yahr scale scores also significantly improved significantly (*p* = 0.025).

**Table 1:** Participant demographics for sample (*n* = 36).

Variable	Average ± STD	Range
T-MoCA	20 ± 1	[18–22]
Age	69 ± 8	[48–84]
Male, Female	19 M, 17F	na
Years since diagnosis	6.6 ± 5.6	[1–21]
Years since first symptom	10.2 ± 7.6	[1.5–29]
LEDD	750.1 ± 477.2	[150–2,198]

F, female; LEDD, Levodopa equivalent daily dose; M, male; T-MoCa, The Telephone Montreal Cognitive Blind Assessment; STD, standard deviation.

**Table 2:** Clinical testing scores.

Variable	OFF		ON		Test statistic	*p*
	Average ± STD	Range	Average ± STD	Range		
Minutes since last dose	1029.4 ± 205.2	[722–1,495]	62.6 ± 2.1	[60–71]	—	—
Total MDS-UPDRS-III score	33.1 ± 10.1	[19–63]	27.2 ± 10.1	[8–52]	*t* = 5.169	<0.001
Tremor subscore	5.0 ± 5.2	[0–21]	2.8 ± 3.5	[0–12]	*Z* = −3.879	<0.001
PIGD subscore	2.2 ± 1.7	[0–8]	1.7 ± 1.6	[0–8]	*Z* = −3.252	0.001
Hoehn & Yahr	II = 30, III = 4, IV = 2	[II–IV]	II = 33, III = 3	[II–III]	*Z* = −2.236	0.025

MDS-UPDRS-III, Movement Disorders Society – Unified Parkinson Disease Rating Scale, part 3; PIGD, postural instability and gait difficulty; STD, standard deviation.

Aside from levodopa, several participants were taking other PD-related medications including: triple-combination medication containing carbidopa, levodopa, and entacapone (*n* = 2), pramipexole (*n* = 5), ropinirole (*n* = 2), amantadine (*n* = 4), and rasagiline (*n* = 1).

### Mean spatiotemporal variables multivariate analysis

Four mean spatiotemporal measures of gait (cadence, velocity, stride length and double limb support) were included in the multivariate model to test if medication and/or cue condition significantly altered the combined multidimensional outcome. At the multivariate level, there was a significant interaction with medium effect (*Λ* = 0.748, *F*(8,134) = 2.620, *p* = 0.011, η_p_^2^ = 0.135). Main effects were also significant at the multivariate level, with a large effect of medication (Λ = 0.463, *F*(4,32) = 9.280, *p* < 0.001, η_p_^2^ = 0.537) and condition (Λ = 0.153, F(8,134) = 26.062, *p* < 0.001, η_p_^2^ = 0.609).

### Cadence

**Figure 1:**
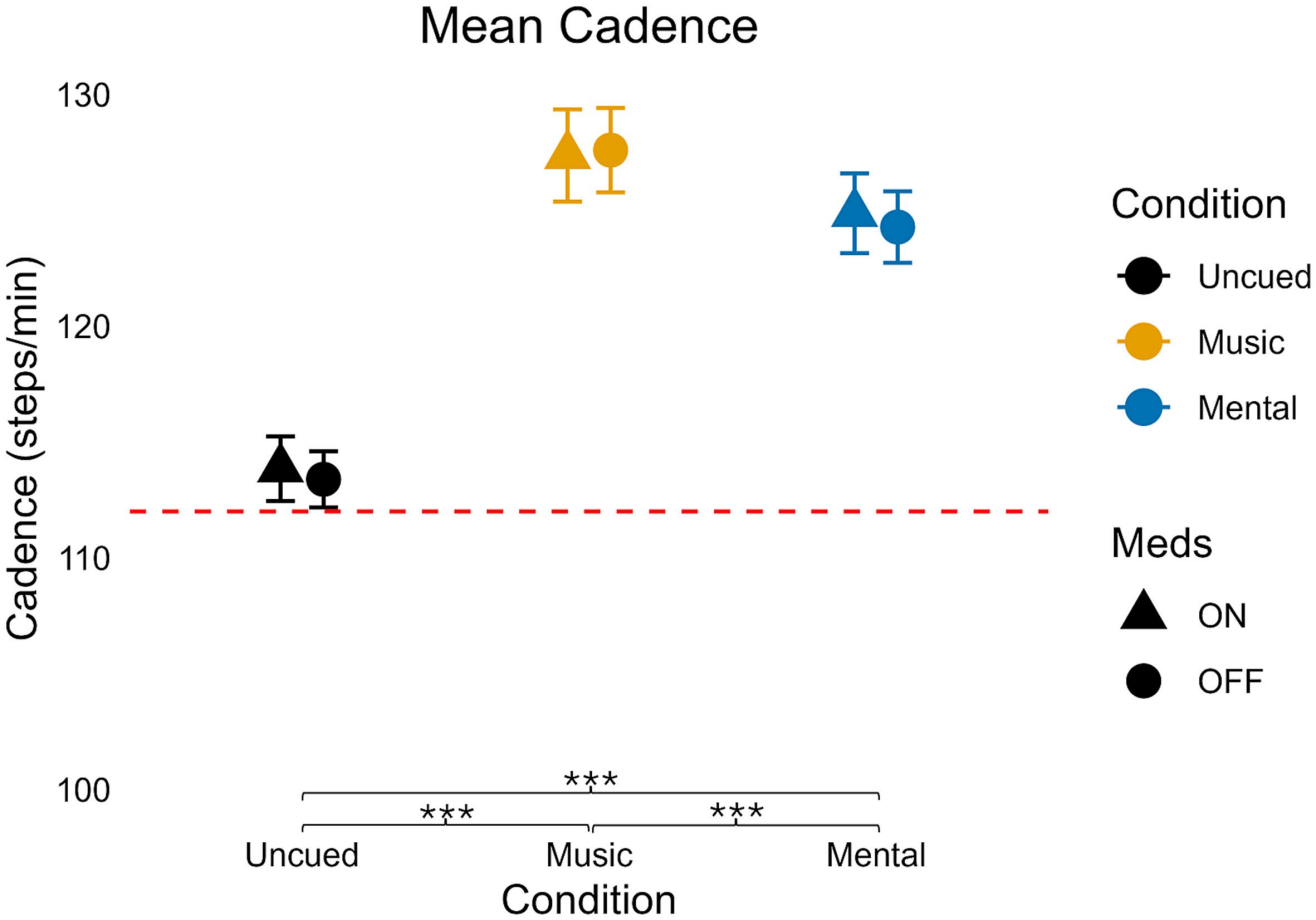
Mean cadence values for medication and condition. Error bars indicate standard error. Horizontal dashed line indicates the average uncued walking data from 28 older adult controls to give additional context as the protocols, equipment, and location of testing were similar (age in years, average ± standard deviation: 68 ± 6) from Harrison et al. (2025). Significant pairwise comparisons are noted as *p < 0.05, **p ≤ 0.01, ***p ≤ 0.001.

For cadence (Table 3; Figure 1), there was no significant interaction (*F*(1.476,51.658) = 1.211, *p* = 0.295, η_p_^2^ = 0.033, small effect). There was no significant main effect of medication on cadence (*F*(1,35) = 0.142, *p* = 0.708, η_p_^2^ = 0.004, less than small effect) but there was a significant, large main effect of cue condition on cadence (*F*(1.326, 46.397) = 104.706, *p* < 0.001, η_p_^2^ = 0.749).

**Table 3:** Gait characteristics (average ± standard deviation).

Variable	OFF			ON		
	Uncued	Mental	Music	Uncued	Mental	Music
Mean						
Cadence (steps/min)	113.39 ± 7.27	124.27 ± 9.24	127.59 ± 10.93	113.84 ± 8.36	124.87 ± 10.34	127.36 ± 11.94
Velocity (m/s)	1.12 ± 0.22	1.27 ± 0.24	1.25 ± 0.25	1.16 ± 0.2	1.33 ± 0.23	1.31 ± 0.24
Stride length (m)	1.18 ± 0.2	1.23 ± 0.21	1.17 ± 0.23	1.22 ± 0.19	1.28 ± 0.21	1.24 ± 0.22
Double limb support (%)	20.46 ± 3.96	18.83 ± 4.2	19.36 ± 4.3	20.23 ± 3.8	18.49 ± 4.15	18.67 ± 4.23
Variability						
Cadence CV (%)	2.07 ± 0.74	2.05 ± 0.45	2.95 ± 1.51	2.08 ± 0.77	2.21 ± 0.96	2.81 ± 1.26
Velocity CV (%)	3.91 ± 1.66	3.37 ± 0.96	4.2 ± 1.65	3.63 ± 1.3	3.58 ± 1.42	3.85 ± 1.73
Stride length CV (%)	3.02 ± 1.52	2.82 ± 1.05	4.29 ± 2.37	2.64 ± 0.99	2.97 ± 1.97	3.98 ± 2.52
Double limb support CV (%)	5.59 ± 1.47	5.95 ± 1.63	6.63 ± 2.36	6.06 ± 2.26	6.9 ± 3.35	7.14 ± 2.81

m, meters; m/s, meters per second; CV, coefficient of variation.

Pairwise comparisons indicated that both cued conditions increased cadence compared to Uncued (Music vs. Uncued: *p* < 0.001, Mental vs. Uncued: *p* < 0.001). The Music condition demonstrated the highest cadence, significantly greater than Mental (*p* < 0.001).

### Velocity

**Figure 2:**
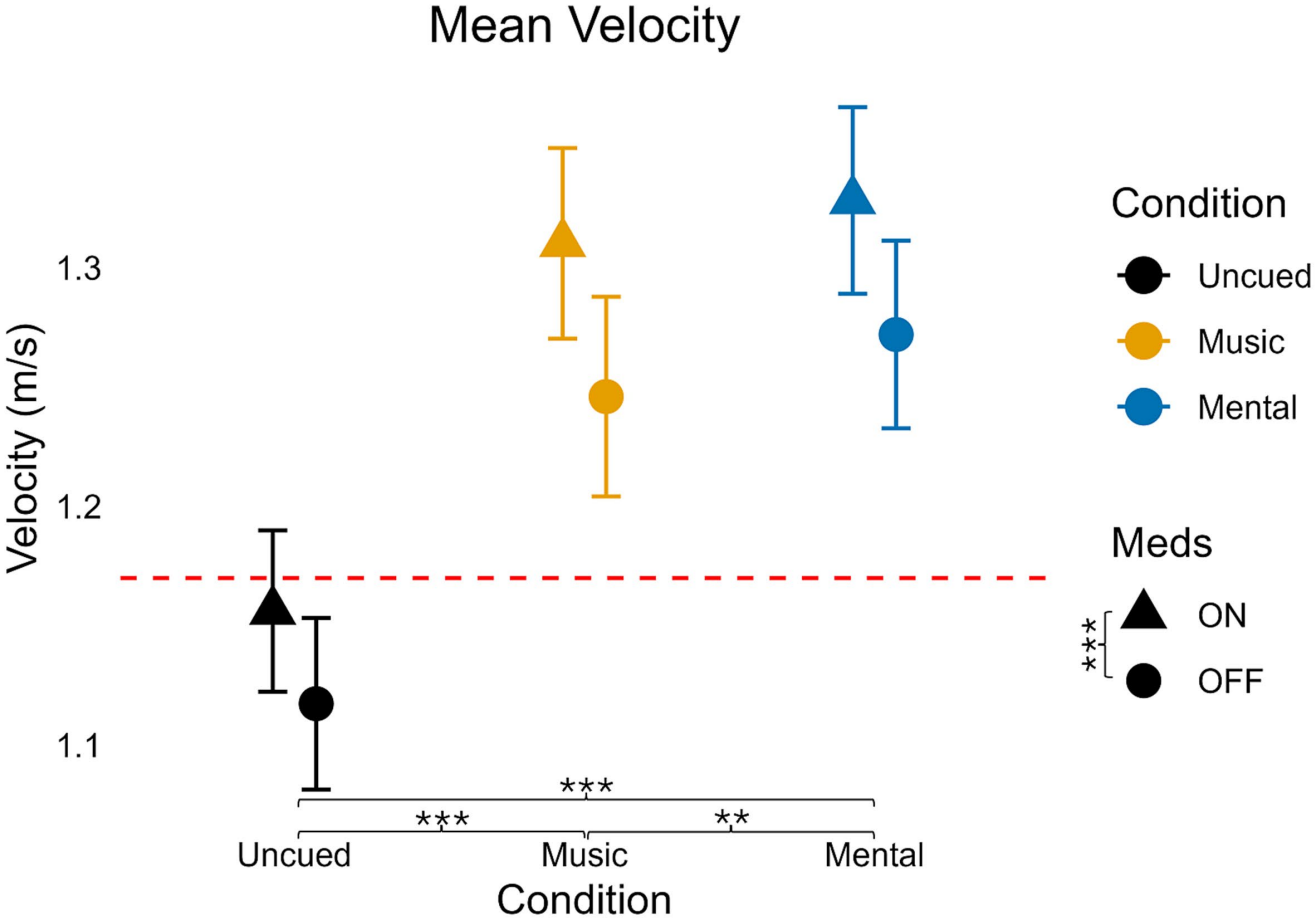
Mean velocity values for medication and condition. Error bars indicate standard error. Horizontal dashed line indicates the average uncued walking data from 28 older adult controls to give additional context as the protocols, equipment, and location of testing were similar (age in years, average ± standard deviation: 68 ± 6) from Harrison et al. (2025). Significant pairwise comparisons are noted as *p < 0.05, **p ≤ 0.01, ***p ≤ 0.001.

For velocity (Table 3; Figure 2), there was no significant interaction (*F*(2,70) = 2.432, *p* = 0.095, η_p_^2^ = 0.065, medium effect). There were significant, large main effects of medication (*F*(1,35) = 18.513, *p* = <0.001, η_p_^2^ = 0.346) and cue condition on velocity (*F*(1.197,41.91) = 73.318, *p* < 0.001, η_p_^2^ = 0.677).

Regardless of cue condition, medication increased velocity (*p* < 0.001). Regardless of medication, there were significant differences between all three cueing conditions. The Mental condition had the greatest velocity, followed by Music and Uncued (Mental vs. Uncued: *p* < 0.001, Mental vs. Music: *p* = 0.006, Music vs. Uncued: *p* < 0.001).

### Stride length

**Figure 3:**
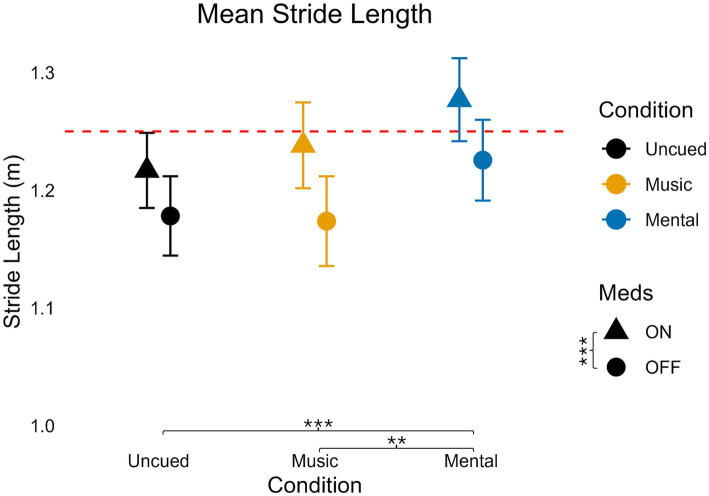
Mean stride length values for medication and condition. Error bars indicate standard error. Horizontal dashed line indicates the average uncued walking data from 28 older adult controls to give additional context as the protocols, equipment, and location of testing were similar (age in years, average ± standard deviation: 68 ± 6) from Harrison et al. (2025). Significant pairwise comparisons are noted as *p < 0.05, **p ≤ 0.01, ***p ≤ 0.001.

For stride length (Table 3; Figure 3), there was no significant interaction (*F*(2,70) = 2.577, *p* = 0.083, η_p_^2^ = 0.069, medium effect). There were significant, large main effects of medication (*F*(1,35) = 27.255, *p* < 0.001, η_p_^2^ = 0.438) and cue condition on stride length (*F*(1.304,45.643) = 10.584, *p* < 0.001, η_p_^2^ = 0.232).

Regardless of cue condition, medication increased stride length (*p* < 0.001). Pairwise comparisons for condition demonstrated that the Mental condition had the greatest stride length (Mental vs. Uncued: *p* < 0.001, Mental vs. Music: *p* < 0.001). There was no significant difference in stride length between Music and Uncued (*p* = 1.0).

### Double limb support percent

**Figure 4:**
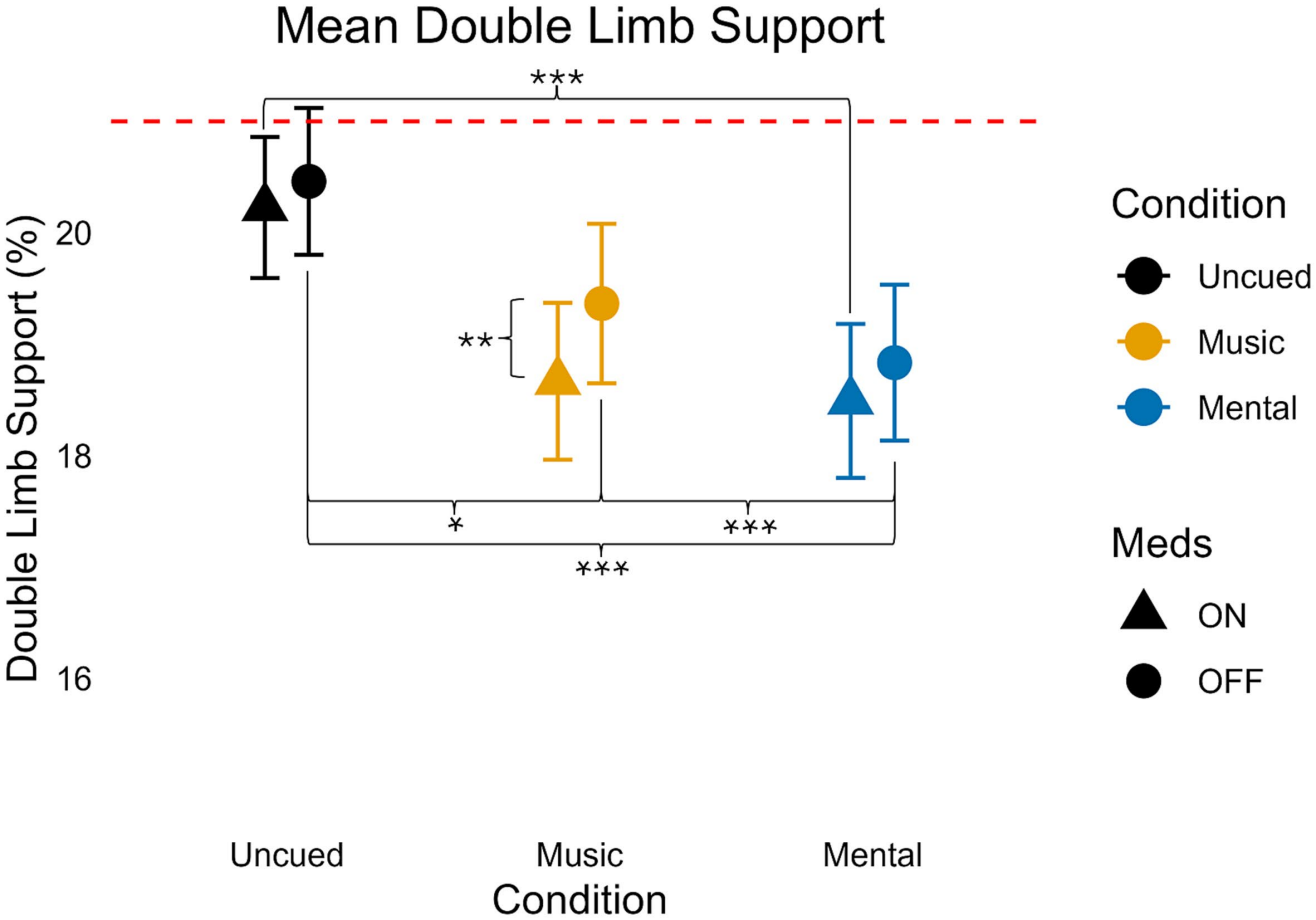
Mean double limb support values for medication and condition. Error bars indicate standard error. Horizontal dashed line indicates the average uncued walking data from 28 older adult controls to give additional context as the protocols, equipment, and location of testing were similar (age in years, average ± standard deviation: 68 ± 6) from Harrison et al. (2025). Significant pairwise comparisons are noted as *p < 0.05, **p ≤ 0.01, ***p ≤ 0.001.

DLS (Table 3; Figure 4) was the only variable that demonstrated a significant interaction between medication and cue condition, with a medium effect (*F*(2,70) = 3.727, *p* = 0.029, η_p_^2^ = 0.096). There were significant, large main effects for medication (*F*(1,35) = 7.096, *p* = 0.012, η_p_^2^ = 0.169) and cue condition (*F*(1.545,54.069) = 53.691, *p* < 0.001, η_p_^2^ = 0.605).

Starting with the pairwise comparison of the main effects, Medication decreased the percentage of time spent in DLS (*p* = 0.012). Regardless of medication, there were significant differences between all three cueing conditions. Mental had the least time spent in DLS compared to Music (*p* = 0.013) and Uncued (*p* < 0.001). Music spent less time in DLS than Uncued (*p* < 0.001).

Considering the interactions of medication and cueing conditions, there was no significant difference in DLS in the Uncued condition between ON and OFF medication (*p* = 0.13) nor was there any significant difference in DLS in the Mental condition between ON and OFF medication (*p* = 0.053). However, during the Music Condition, DLS was significantly reduced when ON as compared to OFF medication (*p* = 0.006).

Considering the interactions of cueing conditions by medication status, DLS was the smallest ON medication during Mental trials, but this was only significantly different when comparing Mental to Uncued (*p* < 0.001). In the ON state, there was no significant difference between Music and Mental cues (*p* = 0.408). In the OFF medication state, there was a significant difference between all three cueing conditions. When participants were OFF medication, DLS was the smallest during Mental, and was significantly different from Uncued (*p* < 0.001) and Music (*p* = 0.013). Participants also had smaller DLS during the Music condition compared to the Uncued condition when OFF medication (*p* < 0.001).

### Variability of spatiotemporal variables multivariate analysis

Four variability spatiotemporal measures of gait (coefficient of variation of cadence, velocity, stride length, and double limb support) were included in the multivariate model to test if medication and/or cue condition significantly altered the combined multidimensional outcome. At the multivariate level, there was not a significant interaction (*Λ* = 0.89, *F*(8,134) = 1.001, *p* = 0.438, η_p_^2^ = 0.056, small effect), indicating that further investigation of interactions at the univariate level would be unnecessary. However, for completeness of results, we have included the non-significant interactions for each individual variable.

Main effects were significant and large at the multivariate level, with an effect of medication (Λ = 0.521, *F*(4,32) = 7.344, *p* < 0.001, η_p_^2^ = 0.479) and condition (Λ = 0.383, F(8,134) = 10.311, *p* < 0.001, η_p_^2^ = 0.363).

### Cadence coefficient of variation

**Figure 5:**
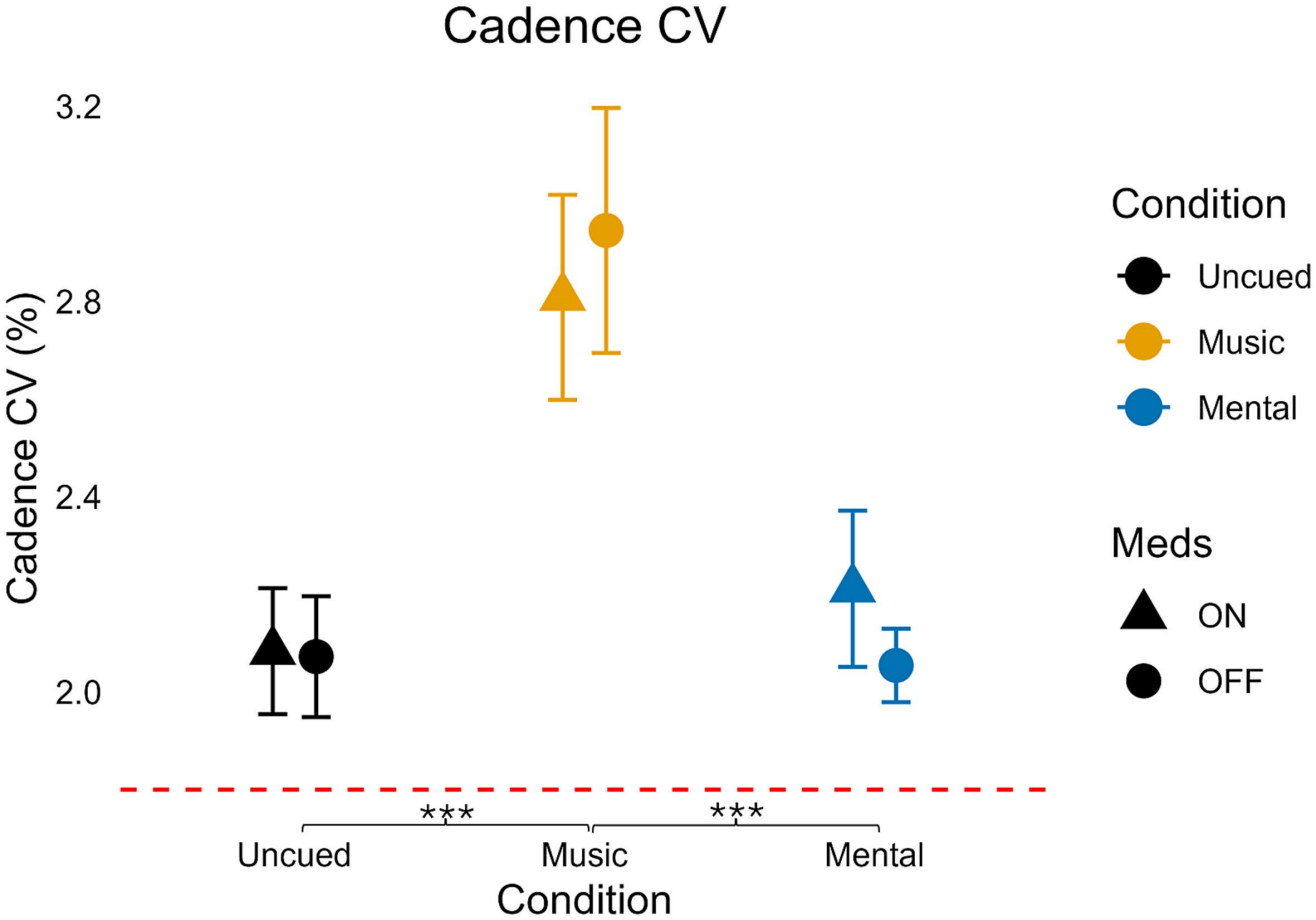
Variability of cadence for medication and condition. Error bars indicate standard error. Horizontal dashed line indicates the average uncued walking data from 28 older adult controls to give additional context as the protocols, equipment, and location of testing were similar (age in years, average ± standard deviation: 68 ± 6) from Harrison et al. (2025). Significant pairwise comparisons are noted as *p < 0.05, **p ≤ 0.01, ***p ≤ 0.001.

As mentioned, there was no significant interaction for cadence CV (*F*(2,70) = 0.665, *p* = 0.517, η_p_^2^ = 0.019, small effect; Table 3; Figure 5). There was no significant main effect of medication on cadence CV (*F*(1,35) = 0.000, *p* = 0.990, η_p_^2^ = 0.000, less than small effect) but there was a significant, large main effect of condition on cadence CV (*F*(1.548, 54.176) = 13.859, *p* < 0.001, η_p_^2^ = 0.284).

Pairwise analysis indicated the Mental condition was not significantly different from the Uncued condition (*p* = 1.0) but that the Music condition had significantly higher cadence CV compared to the Uncued (*p* = 0.001) and Mental (*p* < 0.001) conditions.

### Velocity coefficient of variation

**Figure 6:**
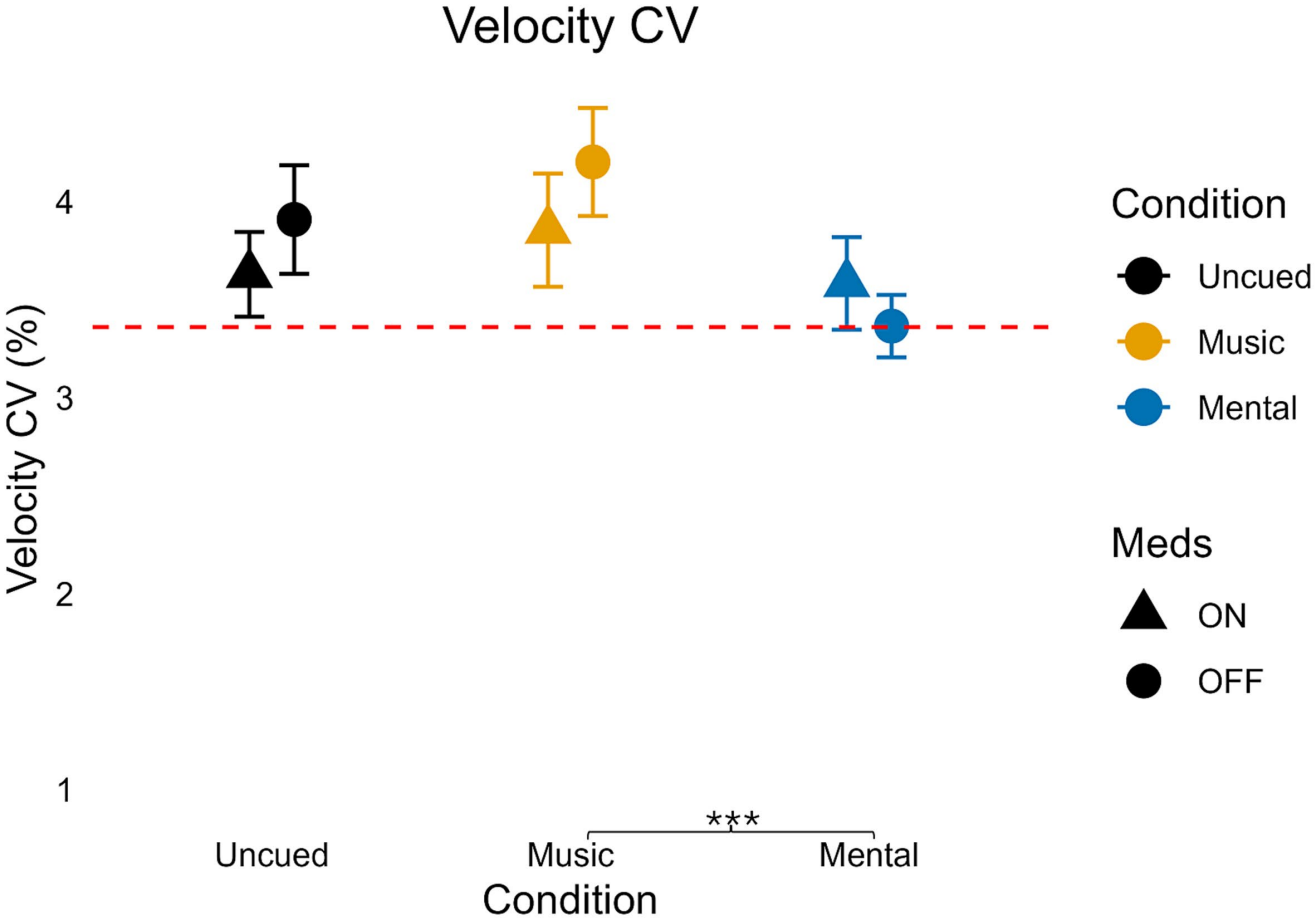
Variability of velocity for medication and condition. Error bars indicate standard error. Horizontal dashed line indicates the average uncued walking data from 28 older adult controls to give additional context as the protocols, equipment, and location of testing were similar (age in years, average ± standard deviation: 68 ± 6) from Harrison et al. (2025). Significant pairwise comparisons are noted as *p < 0.05, **p ≤ 0.01, ***p ≤ 0.001.

There was no significant interaction for velocity CV (*F*(2,70) = 2.722, *p* = 0.073, η_p_^2^ = 0.072, medium effect; Table 3; Figure 6). There was no significant main effect of medication on velocity CV (*F*(1,35) = 1.435, *p* = 0.239, η_p_^2^ = 0.092, medium effect) but there was a significant, large main effect of condition on velocity CV (*F*(1.662, 58.186) = 5.938, *p* = 0.007, η_p_^2^ = 0.145).

Pairwise analysis indicated neither the Mental condition nor the Music condition was significantly different from the Uncued condition (Mental vs. Uncued: *p* = 0.201), (Music vs. Uncued: *p* = 0.557). When comparing cued conditions, velocity CV was significantly higher in Music compared to Mental (*p* < 0.001).

### Stride length coefficient of variation

**Figure 7:**
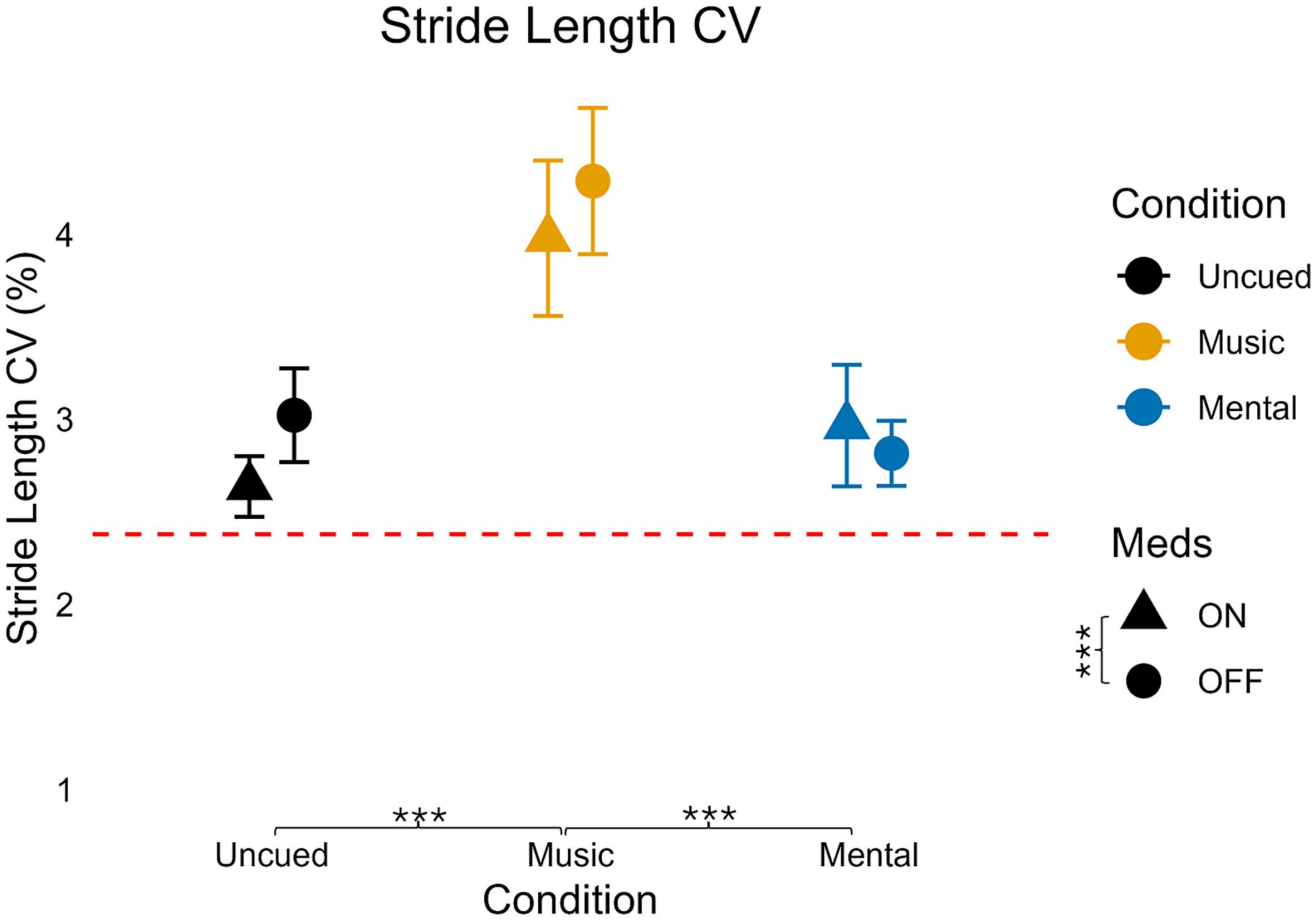
Variability of stride length for medication and condition. Error bars indicate standard error. Horizontal dashed line indicates the average uncued walking data from 28 older adult controls to give additional context as the protocols, equipment, and location of testing were similar (age in years, average ± standard deviation: 68 ± 6) from Harrison et al. (2025). Significant pairwise comparisons are noted as *p < 0.05, **p ≤ 0.01, ***p ≤ 0.001.

For stride length CV (Table 3; Figure 7), there was no significant interaction (*F*(2,70) = 1.759, *p* = 0.18, η_p_^2^ = 0.048, small effect). There was no significant main effect of medication on stride length CV (*F*(1,35) = 3.554, *p* = 0.068, η_p_^2^ = 0.092, medium effect) but there was a significant, large main effect of condition on stride length CV (*F*(1.455,50.942) = 26.429, *p* < 0.001, η_p_^2^ = 0.430).

Similar to cadence CV, pairwise analysis indicated stride length CV in the Mental condition was not significantly different from the Uncued condition (*p* = 1.0). Stride length CV was significantly higher in the Music condition compared to the Uncued (*p* < 0.001) and Mental (*p* < 0.001) conditions.

### DLS coefficient of variation

**Figure 8:**
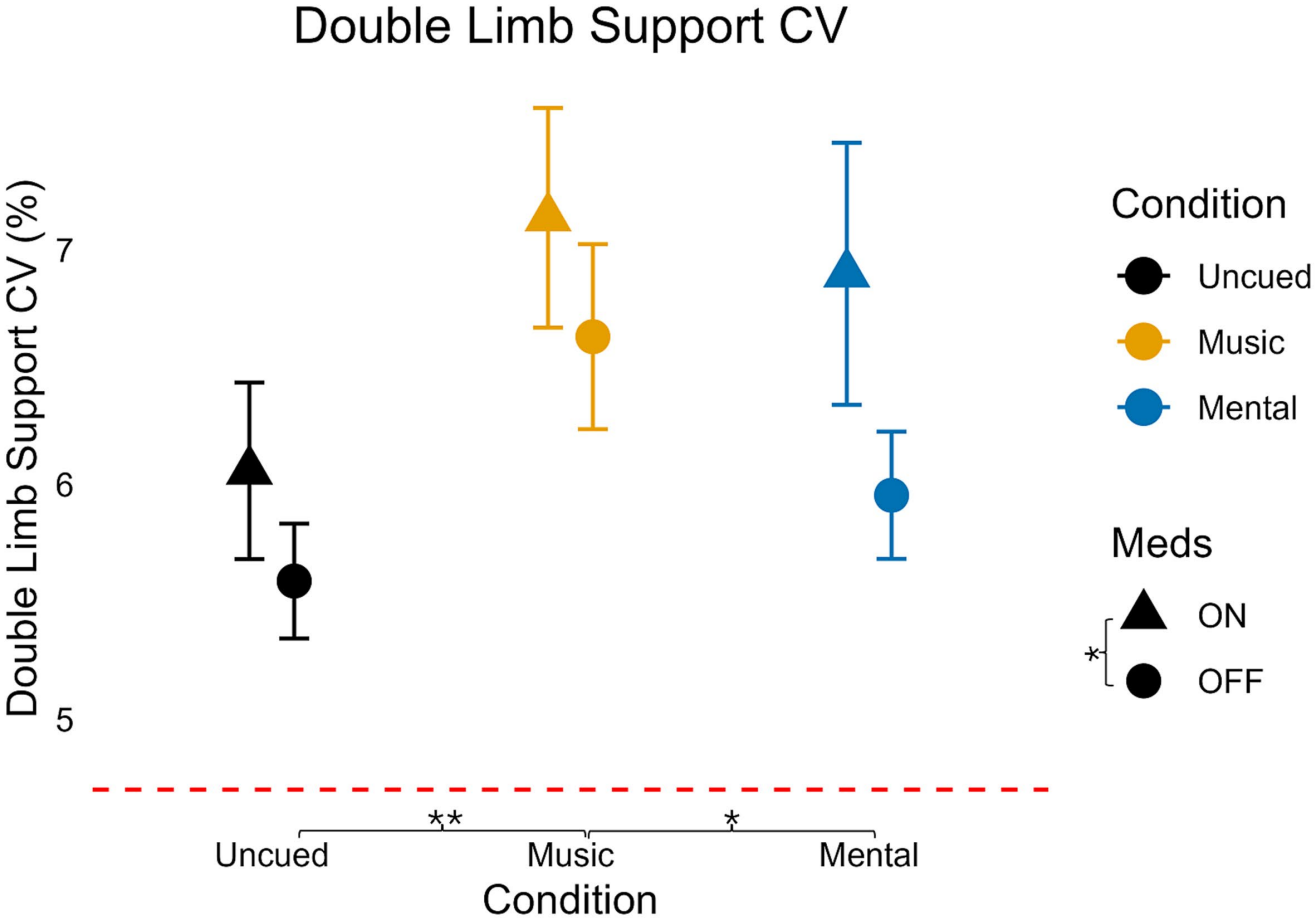
Variability of double limb support for medication and condition. Error bars indicate standard error. Horizontal dashed line indicates the average uncued walking data from 28 older adult controls to give additional context as the protocols, equipment, and location of testing were similar (age in years, average ± standard deviation: 68 ± 6) from Harrison et al. (2025). Significant pairwise comparisons are noted as *p < 0.05, **p ≤ 0.01, ***p ≤ 0.001.

For DLS CV (Table 3; Figure 8), there was no significant interaction (*F*(2,70) = 0.272, *p* = 0.763, η_p_^2^ = 0.008, less than small effect). There was a significant, medium main effect of medication on DLS CV (*F*(1,35) = 4.356, *p* = 0.044, η_p_^2^ = 0.111) and there was a significant, large main effect of condition (*F*(1.405,49.167) = 7.877, *p* = 0.003, η_p_^2^ = 0.537, η_p_^2^ = 0.184).

For the pairwise analysis of medication, DLS CV was lower when OFF medication compared to ON medication (*p* = 0.044).

Pairwise analysis across conditions indicated the Mental condition was not significantly different from the Uncued condition (*p* = 0.192) but that DLS CV was significantly higher in the Music condition compared to the Uncued (*p* = 0.008) and Mental (*p* = 0.011) conditions.

## Discussion

While auditory cueing, including music-based cueing, is recommended for individuals with PD, there are still many unknowns about the exact mechanism of action, as well as the best ways to implement cues in daily life (Osborne et al., 2022). As daily intake of medication, including levodopa, remains the gold-standard treatment for PD, it is important to understand the effects that medication may have on the ability to utilize various cueing strategies. Our purpose was to investigate the effects of anti-PD medication on walking among people with PD using external music cues and internal mental singing cues. We demonstrate that internal, mental singing cues can be used to improve gait performance, including velocity and variability, in both the ON and OFF medications states and may provide greater benefit than external, music-based cues regardless of medication status.

### Effect of medication

Basal ganglia dysfunction in PD impairs multiple aspects of motor control, as the regions responsible for dopamine production fails to produce adequate neurotransmitters for proper movement. Hypokinesia, or the reduction of movement amplitude, is a key feature observed across multiple motor tasks including writing (McLennan et al., 1972; Thomas et al., 2017), reaching (Fasano et al., 2022), and walking (Bonacina et al., 2024). In gait, hypokinesia manifests as reductions in stride or step length (Morris et al., 1994). The reduction of dopamine in the basal ganglia lowers output from the thalamus to motor cortex regions, ultimately resulting in reduced activation at the level of the muscle. Anti-parkinsonian medication, mostly consisting of levodopa replacement medication, has been the first line treatment since its inception (Lees et al., 2023). When medication is working well, often labelled as being in the “ON” state, the addition of levodopa through medication helps increase motor output, improving muscle activation during walking (Cioni et al., 1997; Caliandro et al., 2011; Zhao et al., 2025). Changes in muscle activation allow individuals to increase the range of motion and power generation across lower limb joints (Albani et al., 2014; Baudendistel et al., 2021), resulting in larger steps as reflected in the present sample and similar to Rochester et al. (2011) and Smulders et al. (2016).

While >80% of individuals with PD are prescribed at least one anti-parkisonian medication (Dahodwala et al., 2017), less than 30% of patients rate their PD medications as good or very good (Jost and Bausch, 2017). This discordance may be, in part, due to the fact that not all aspects of motor control are improved on medication (Antonini et al., 2023), including some aspects of motor timing (Pasetti et al., 2003; Jones et al., 2011; Curtze et al., 2015). Like amplitude of movement, the timing domain of movement is also impaired by reductions in dopamine, even beyond the decreased internal velocity of timekeeping related to reductions in amplitude (Artieda et al., 1992; Dušek et al., 2012). Timing deficits are less understood than amplitude deficits, potentially due to the complex neurophysiological mechanisms involved in explicit and implicit timing mechanisms (Coull et al., 2011; Yin, 2014; Lee et al., 2018). These complex mechanisms may contribute to the mixed results reported across studies for cadence, DLS, and variability between medication states (Jones et al., 2008; Cameron et al., 2016; Bernardinis et al., 2019). We observed, like others (Blin et al., 1991; O’Sullivan et al., 1998; Almeida et al., 2007; Bryant et al., 2011b; Rochester et al., 2011; Barbosa et al., 2024), that cadence and cadence variability are relatively unaffected by medication. In the present study, cadence was nearly identical between ON and OFF states within each cueing condition. We hypothesize that rhythm-based cueing paradigms directly target timing deficits that are not treated effectively with anti-PD medication. As medication effectiveness may fluctuate throughout the day (Devraj et al., 2024), deploying compensation techniques such as rhythmic-based cueing may support mobility in times of need, such as end-of-dose wearing off. Specifically, improving rhythm through the use of RAC can enhance both spatial and temporal measures of gait, beyond what is gained with anti-PD medication alone. However, for purposes of implementation, “improving” rhythm may not necessarily align with increasing cadence. Harrison et al. (2025) demonstrated that the cadence that elicited the longest stride length or fastest gait velocity is not always the highest cadence value. Future studies should further investigate the relationship between cadence and stride length specific to interventional efforts, including medication and physical rehabilitation techniques.

For measures of variability, cadence CV, velocity CV, and stride length CV were not statistically altered by medication. This was similar to the findings of Erra et al. (2019), in which cadence CV, velocity CV, and stride length CV were not significantly different between ON and OFF medication states. However, DLS CV in our sample was consistently higher in the ON state compared to OFF state regardless of the cue condition. Our results may align with previous research indicating dynamic balance control, measured with DLS mean and variability, may be impaired with the addition of levodopa (Gabell and Nayak, 1984; Yogev et al., 2005). Our sample had relatively lower mean DLS and lower DLS CV in the OFF state compared to prior studies (Bryant et al., 2011a, 2016; Lord et al., 2011; Rochester et al., 2011). These results may indicate that participants in the present sample had greater dynamic control during gait than participants in other similar studies. In a sample in which DLS and DLS CV is low, anti-PD medication may allow participants to alter their control during gait, potentially exploring the bounds of balance during walking. This exploration could be reflected as increased variability (Sternad, 2018). It may be that anti-PD medication only decreases DLS CV if dynamic control of gait is substantially impaired.

### Effect of condition

Rhythmic auditory cueing represents a well utilized compensation strategy to overcome gait impairment, including those related to medication issues like wearing off (Nonnekes et al., 2019). Nonnekes et al. (2019) explains many compensation techniques may provide adequate relief for declines in gait automaticity when deployed appropriately. Specifically, these techniques may allow patients to perform more “goal-directed gait control”. Music-based cues are classified as an “external cue” compensation strategy, as an external stimulus provides a movement target. Mental-singing cues may be considered a mixed methods approach, capitalizing on the self-initiated “internal cue” but using it in a continuous manner like “external cues.” As patients have described internal cueing most useful during gait initiation compared to other aspects of gait (Tosserams et al., 2020), the addition of the musical aspect of the mental singing cue may increase usage and efficacy compared to pure external cues or internal cues alone.

Both the Music and Mental conditions focus on cadence as the primary outcome, directly targeting rhythmicity of gait. For cadence, the Music condition demonstrated the highest cadence, significantly higher than the Mental condition, replicating the basic findings from our previous studies (Harrison et al., 2018, 2019, 2025) in a new sample. While the difference between Music and Mental was statistically significant, the trials, regardless of condition, only differed by 2% or approximately 3 steps/min. The change between Uncued and Mental of 11 steps/min or 9%, is considerably larger. For stride length, only mental singing statistically increased stride length compared to Uncued. The increase of stride length by 5 cm for mental singing without medication was larger than the 4 cm increase in stride length with medication. This is compared to the non-significant difference of stride length comparing Uncued to Music, in which stride length was reduced by 1 cm during the Music trials compared to the Uncued trials in the OFF state. Haussler et al. (2026) reported similar results when comparing Music to Mental singing and hypothesized that different strategies may underlie walking to different cueing types. For Music conditions, participants may be more likely to focus on matching cadence as they continuously update their motor plan to match the beat. Participants during the Music condition may default to uncued stride length or reduce their stride length as their attention is directed to synchronizing to an external source. Importantly, listening to music without explicit instructions does not alter gait in people with PD (Ready et al., 2025), inferring that the instruction to synchronize in conjunction with the external music may provide distinctive challenges as related to attention. For Mental conditions, there is no external rhythm to repeatedly adjust to and the act of mental singing, instead of passive listening, may tap into the benefits to motor tasks that arise from the production of music (Pacchetti et al., 2000; Dunbar et al., 2012). As “imagined” musical cues produce greater motor network activation than heard cues (Schaefer et al., 2014), mental singing may intrinsically promote greater stride length than music cues alone. When evaluating the effects of cadence and stride length combined, both Music and Mental singing cueing conditions increased walking velocity. Although the Mental condition had significantly greater velocity than the Music condition, the difference between cueing types did not meet the threshold for minimal clinically important difference (MCID) for velocity (0.082 m/s) potentially indicating that both may be viable forms of rhythmic cueing (Baudendistel et al., 2024). Targeting cue type to each individual’s deficits, either reduced cadence or reduced stride length, or to their preference may be beneficial if gait velocity is the primary targeted outcome.

While there are potential differential effects for cadence and stride length based on the type of cue employed, mental singing appears to be favored compared to external music as a cueing technique when integrating the results of variability and dynamic stability. Primarily, participants walked with significantly higher variability for all gait measures in the Music condition than the Mental condition, potentially indicating worse stability during Music trials. As previously discussed in Harrison et al. (2019), the Music cue, when compared to the Mental condition, may align closer to what is observed during a dual-tasking paradigm, where increases of variability are commonly observed. Further, during the Mental cue, variability was not statistically different than Uncued walking for all gait measures, even when mean values of cadence, velocity, and stride length were increased. These results also match our previous work (Harrison et al., 2018, 2019, 2025). Interestingly, there is conflicting evidence on whether variability of gait is velocity-dependent. Frenkel-Toledo et al. (2005) identified that faster walking velocity is associated with reductions in rhythm variability while Bryant et al. (2011a) found no association between faster walking velocity and rhythm variability. The contrasting results between Music and Mental cues further complicate the story of the relationship between gait velocity and variability among people with PD. While traditionally higher variability is considered an indicator of worse stability and, therefore, worse gait performance, higher variability is expected during the initial stages of motor learning (Dhawale et al., 2017). Investigating whether repeated practice of the Music cues reduces variability over time may be relevant to individuals who prefer the external musical cue for implementation. Moreover, while linear variability, such as CV, is a more common outcome for gait assessments in people with PD compared to non-linear variability, linear variability only provides the mean amplitude of stride-to-stride fluctuations. Non-linear variability, which helps describe the temporal changes in variability, may provide greater insight into gait dynamics of PD (Hausdorff, 2009; Lukšys and Griškevičius, 2021). For example, in Lheureux et al. (2020) rhythmic auditory stimulation did not alter linear variability in people with PD, but changes to non-linear variability calculated with averaged Detrended Fluctuation Analysis were apparent. Investigating non-linear dynamics between Music and Mental cues could improve our knowledge of how RAC alters gait variability.

### Interaction effect

A pivotal aspect of this research was to investigate the effect of the two types of RAC, Music-based cues or Mental singing-based cues, in the context of an individual’s medication status. Our previous work repeatedly demonstrated both Music and Mental singing cues improve gait velocity and Mental cues reduce variability compare to Music cues (Harrison et al., 2018, 2019, 2025). Yet these studies were all conducted in the “ON” state. It is not known if medication status affects the ability of participants with PD to utilize either Music or Mental cues. In the study herein, DLS percent was the only variable to have an interaction effect between cue condition and medication. Mean DLS changed with cueing (Music and Mental) depending on whether the person was ON or OFF their medication. For all the other gait measures (velocity, stride length, cadence, variability), the effects of cueing were the same regardless of medication status.

DLS was selected to provide a measure of dynamic stability during gait, similar to Almeida et al. (2007) and Rinaldi et al. (2014). Mean DLS is a measure of time spent with both feet on the ground during a stride. More time spent with both feet on the ground, results in a wider base of support and thus a more conservative gait pattern (Gwerder et al., 2025). Individuals with earlier stages of PD walk with less DLS than those in more advanced stages (Hass et al., 2012), indicating that reducing double support could be considered an improvement in dynamic stability of gait. There was a significant main effect of medication, indicating that medication reduced DLS regardless of the cueing condition. In each cueing condition, DLS mean was reduced compared to Uncued gait, albeit not significantly between the Uncued and Mental singing conditions. While previous ON versus OFF gait analyses reported significant reductions of mean DLS between 10% (Bryant et al., 2011b) and 20% (Bowes et al., 1990), the mean DLS values for these studies were much higher. For example, in Bryant et al. (2011b), participants in the OFF medication state walked with a DLS mean of 35%. In the present study, participants walked with 20% DLS. Even compared to healthy older adults, our sample of individuals with PD in the OFF state walked with considerably less mean DLS (Healthy older males age 70–74, DLS mean and standard deviation 26.3% ± 3%; Hollman et al., 2011). These values of DLS were not expected given the traditional explanation that less time spent in double support represents “less conservative” gait and potentially better dynamic stability in our group of individuals with PD compared to the healthy older adults (Hollman et al., 2011). Our sample more closely aligns with Curtze et al. (2015) who used similar equipment to the study herein, whose sample walked with 22% mean DLS and that study reported no difference between ON and OFF medication states. There may be a floor effect, in which participants with relatively lower mean DLS OFF medications cannot further reduce DLS with medication. Alternatively, small changes in DLS may mirror the non-significant changes observed in the temporal domain (i.e., cadence). As the cueing conditions significantly reduced mean DLS compared to Uncued trials, regardless of medication state, DLS may be a dopa-resistant feature that can be mitigated by RAC, especially Mental cueing.

The Mental conditions demonstrated the lowest mean DLS as compared to the Music or Uncued trials. While medication did not influence DLS during Uncued gait nor the effectiveness of the Mental cue, there was a significant difference during the Music conditions between ON- and OFF-medication states. We hypothesize that in the OFF-medication state, individuals with PD could not effectively utilize the rhythmic cue to the level that they could when ON medication. This may be because Music cueing requires constant integration of auditory information, while Mental singing does not. As anti-PD medication may assist in reducing reaction time during the stimulus preprocessing stage in both individuals with PD (Onder et al., 2025) and otherwise healthy adults (Rihet et al., 2002), the addition of anti-PD medication may assist in sensory processing during MUSIC cues. The lack of dopamine in the OFF state may not affect DLS during the mental singing task, as no external auditory processing is needed. Alternatively, since medication status was not randomized, the difference observed between conditions may be an effect of repeated exposure to the task during the ON trials. However, DLS during the Mental condition remained lower between ON and OFF medication tests. If motor learning is a factor in cueing paradigms, it is not apparent during the mental singing trials. As mentioned, further investigating novelty of task and repeated bouts of Music versus vs. Mental singing would be important in the future.

## Limitations

The primary limitation of the current study is that medication state was not randomized. While completing assessments first OFF and then ON within a single day is common (Smulders et al., 2016), it does introduce bias, as every individual in the ON medication state had already performed the tasks previously. When compared to other studies investigating the effect of medication changes on RAC, only Rochester et al. randomized medication status, having participants come into the lab twice, 2 weeks apart (Rochester et al., 2011). It is difficult to compare the cues between our tasks and theirs, as their internal cue was “to take big steps” and their external cue was set at 100% of preferred cadence, while ours was set at 120%. However, the effects of medication during the uncued trials were relatively similar, as medication increased velocity and stride length but did not increase cadence.

There are several measures that would be useful to collect in future studies. First, we did not measure synchronization within or between trials. Our study was set up to investigate whether gait performance metrics change while completing the cued conditions. As research suggests synchronization to the beat is impaired for people with PD (Bieńkiewicz and Craig, 2015) and the ability to synchronize to music may impact the effectiveness of RAC (Cochen De Cock et al., 2018), future studies should investigate how well participants can complete the task in terms of synchronization. It is also possible that participants potentially increased or decreased their pace during the trials. Haussler et al. (2026) found no significant difference between the first 10 s and the last 10 s for mean velocity for either the Music cue or Mental singing cue. While effect sizes were trivial, stride length significantly decreased over time and cadence decreased during the mental singing cue. Third, attention was not measured directly or indirectly in this study. In both conditions, participants were provided similar instructions. These instructions inherently focused their attention on the task at hand. As variability increased during the Music condition, the increases in variability observed while listening to music may provide different attention-related challenges than mental signing. As listening to music without instruction does not alter gait (Ready et al., 2025), there is a component of directed attention in the context of external or internally generated music that should be explored. Another important limitation is the relatively mild degree of PD observed in our sample. On average our participants were considered as having “moderate” severity in the OFF medication state, and “mild severity” in the ON state (Martínez-Martín et al., 2015). Most participants had a Hoehn and Yahr score of II, indicating “no impairment of balance.” Even the PIGD subscore was relatively low, averaging about 2 out of a possible score of 12. Beyond clinical measures of PD severity, mild gait impairment was also observed, as seen with an average gait velocity of within one standard deviation of normative values for healthy older adults (Hollman et al., 2011) and unexpectedly low values of DLS. This may be in part due to the inherent nature of the requirements of the study as participants needed to be able to tolerate medication withdrawal. We could have incidentally excluded individuals who have worse disease severity or benefit more from medication and were unable or unwilling to complete the withdrawal. As multiple factors, including disease progression, may affect the responsiveness to cues (Lirani-Silva et al., 2019; Baudendistel and Earhart, 2025), a more heterogenous sample may produce different results.

One of the primary goals of this study was to understand if mental singing cues are still useful during times when participants are not optimally medicated. However, we did not test the effect of cues throughout the medication cycle. As walking can vary over the course of a medication cycle (MacKay-Lyons, 1998; Barbosa et al., 2024; Promsri and Federolf, 2025), testing gait throughout the day would be of particular relevance, enhancing ecological validity. Newer technologies such as wearable sensors (Brognara et al., 2019) would allow for a better understanding of how individuals use cueing strategies in the real-world where medications fluctuations are common.

Freezing of gait is also affected by medication and timing of medication. We did not exclude freezers from the present study, but no freezing episodes occurred during the walking trials. As we have previously noted that freezers are able to utilize cueing similarly to non-freezers (Horin et al., 2020), and other studies demonstrate that freezers are able to utilize cues both ON and OFF medication (Barthel et al., 2018; Zoetewei et al., 2024), we anticipate that the present results would generalize to individuals who experience freezing episodes.

## Conclusion

As medication effectiveness may wane throughout the day, we investigated the interaction of medication and cueing rehabilitation strategies to better understand whether music and mental cueing strategies are still effective in times when medications are not. Both Music and Mental cue types enhanced gait compared to uncued trials, regardless of medication state, but mental singing cues produced the best results in gait performance including stride length and all measures of variability. Our results demonstrate that rhythmic auditory cueing, including internally-based mental singing and externally-based music, is a feasible and robust strategy that improves gait even when medication is withdrawn.

## Data Availability

The raw data supporting the conclusions of this article will be made available by the authors, upon reasonable request and in keeping with permissions outlined in the IRB protocol and consent forms.
