# Supplementary material for: Effects of anti-parkinsonian medication on gait during internal and external rhythmic auditory cueing in PD
Source: Front Hum Neurosci. 2026 Apr 22;20:1808526. doi: 10.3389/fnhum.2026.1808526 (PMC13144043; doi:10.3389/fnhum.2026.1808526)
Supplement: Supplementary file 1 [file Table_1.docx]

**Supplementary Data A: Estimated Marginal Means for MANOVA 1 (Mean Values of Cadence, Velocity, Stride Length, Double Limb Support %)**

**Grand Means**

| Measure | Mean | Std. Error | 95% Confidence Interval | |
| --- | --- | --- | --- | --- |
|  |  |  | Lower Bound | Upper Bound |
| Cadence | 121.886 | 1.474 | 118.893 | 124.879 |
| Velocity | 1.238 | 0.037 | 1.164 | 1.313 |
| Stride Length | 1.218 | 0.034 | 1.15 | 1.287 |
| Double Limb Support % | 19.341 | 0.671 | 17.978 | 20.703 |

**Effect of Medication**

| Measure | Medication | Mean | Std. Error | 95% Confidence Interval | |
| --- | --- | --- | --- | --- | --- |
|  |  |  |  | Lower Bound | Upper Bound |
| Cadence | Off | 121.75 | 1.431 | 118.846 | 124.655 |
|  | On | 122.022 | 1.6 | 118.773 | 125.272 |
| Velocity | Off | 1.212 | 0.038 | 1.135 | 1.289 |
|  | On | 1.265 | 0.037 | 1.19 | 1.339 |
| Stride Length | Off | 1.192 | 0.034 | 1.123 | 1.262 |
|  | On | 1.244 | 0.034 | 1.175 | 1.313 |
| Double Limb Support % | Off | 19.554 | 0.683 | 18.166 | 20.941 |
|  | On | 19.128 | 0.668 | 17.771 | 20.484 |

**Pairwise Comparison of Medication**

| Measure | Mean Difference | Std. Error | Sig.^b^ | 95% Confidence Interval for Difference^b^ | |
| --- | --- | --- | --- | --- | --- |
|  |  |  |  | Lower Bound | Upper Bound |
| Cadence | -0.272 | 0.722 | 0.708 | -1.739 | 1.194 |
| Velocity | -.053* | 0.012 | <.001 | -0.078 | -0.028 |
| Stride Length | -.051* | 0.01 | <.001 | -0.072 | -0.031 |
| Double Limb Support % | .426* | 0.16 | 0.012 | 0.101 | 0.75 |
| Based on estimated marginal means | | | | | |
| * The mean difference is significant at the .05 level. | | | | | |
| b Adjustment for multiple comparisons: Bonferroni. | | | | | |

**Effect of Cue Condition**

| Measure | Cue | Mean | Std. Error | 95% Confidence Interval | |
| --- | --- | --- | --- | --- | --- |
|  |  |  |  | Lower Bound | Upper Bound |
| Cadence | Uncued | 113.614 | 1.257 | 111.063 | 116.166 |
|  | Mental | 124.569 | 1.598 | 121.324 | 127.814 |
|  | Music | 127.476 | 1.846 | 123.728 | 131.223 |
| Speed | Uncued | 1.137 | 0.034 | 1.067 | 1.206 |
|  | Mental | 1.3 | 0.039 | 1.221 | 1.378 |
|  | Music | 1.278 | 0.04 | 1.197 | 1.359 |
| Stride Length | Uncued | 1.197 | 0.032 | 1.132 | 1.263 |
|  | Mental | 1.251 | 0.034 | 1.181 | 1.321 |
|  | Music | 1.206 | 0.037 | 1.132 | 1.28 |
| Double Limb Support % | Uncued | 20.344 | 0.642 | 19.04 | 21.648 |
|  | Mental | 18.662 | 0.69 | 17.26 | 20.064 |
|  | Music | 19.016 | 0.701 | 17.593 | 20.438 |

**Pairwise Comparison of Cue Condition**

| Measure | (I) Cue | (J) Cue | Mean Difference (I-J) | Std. Error | Sig.^b^ | 95% Confidence Interval for Difference^b^ | |
| --- | --- | --- | --- | --- | --- | --- | --- |
|  |  |  |  |  |  | Lower Bound | Upper Bound |
| Cadence | Uncued | Mental | -10.954* | 0.97 | <.001 | -13.393 | -8.516 |
|  |  | Music | -13.861* | 1.299 | <.001 | -17.128 | -10.595 |
|  | Mental | Uncued | 10.954* | 0.97 | <.001 | 8.516 | 13.393 |
|  |  | Music | -2.907* | 0.659 | <.001 | -4.564 | -1.25 |
|  | Music | Uncued | 13.861* | 1.299 | <.001 | 10.595 | 17.128 |
|  |  | Mental | 2.907* | 0.659 | <.001 | 1.25 | 4.564 |
| Velocity | Uncued | Mental | -.163* | 0.016 | <.001 | -0.204 | -0.122 |
|  |  | Music | -.141* | 0.018 | <.001 | -0.187 | -0.095 |
|  | Mental | Uncued | .163* | 0.016 | <.001 | 0.122 | 0.204 |
|  |  | Music | .022* | 0.007 | 0.006 | 0.006 | 0.038 |
|  | Music | Uncued | .141* | 0.018 | <.001 | 0.095 | 0.187 |
|  |  | Mental | -.022* | 0.007 | 0.006 | -0.038 | -0.006 |
| Stride Length | Uncued | Mental | -.054* | 0.013 | <.001 | -0.085 | -0.022 |
|  |  | Music | -0.008 | 0.016 | 1 | -0.049 | 0.032 |
|  | Mental | Uncued | .054* | 0.013 | <.001 | 0.022 | 0.085 |
|  |  | Music | .045* | 0.008 | <.001 | 0.026 | 0.064 |
|  | Music | Uncued | 0.008 | 0.016 | 1 | -0.032 | 0.049 |
|  |  | Mental | -.045* | 0.008 | <.001 | -0.064 | -0.026 |
| Double Limb Support % | Uncued | Mental | 1.682* | 0.195 | <.001 | 1.191 | 2.173 |
|  |  | Music | 1.329* | 0.191 | <.001 | 0.849 | 1.808 |
|  | Mental | Uncued | -1.682* | 0.195 | <.001 | -2.173 | -1.191 |
|  |  | Music | -.354* | 0.116 | 0.013 | -0.645 | -0.062 |
|  | Music | Uncued | -1.329* | 0.191 | <.001 | -1.808 | -0.849 |
|  |  | Mental | .354* | 0.116 | 0.013 | 0.062 | 0.645 |
| Based on estimated marginal means | | | | | | | |
| * The mean difference is significant at the .05 level. | | | | | | | |
| b Adjustment for multiple comparisons: Bonferroni. | | | | | | | |

**Interaction Effect Medication * Cue Condition**

| Estimates |  |  |  |  |  |  |
| --- | --- | --- | --- | --- | --- | --- |
| Measure | Medication | Cue | Mean | Std. Error | 95% Confidence Interval | |
|  |  |  |  |  | Lower Bound | Upper Bound |
| Cadence | Off | Uncued | 113.387 | 1.211 | 110.929 | 115.846 |
|  |  | Mental | 124.272 | 1.541 | 121.145 | 127.4 |
|  |  | Music | 127.591 | 1.821 | 123.894 | 131.288 |
|  | On | Uncued | 113.841 | 1.393 | 111.013 | 116.67 |
|  |  | Mental | 124.865 | 1.724 | 121.366 | 128.365 |
|  |  | Music | 127.361 | 1.989 | 123.322 | 131.399 |
| Velocity | Off | Uncued | 1.117 | 0.036 | 1.044 | 1.19 |
|  |  | Mental | 1.272 | 0.039 | 1.192 | 1.352 |
|  |  | Music | 1.246 | 0.042 | 1.161 | 1.331 |
|  | On | Uncued | 1.156 | 0.034 | 1.088 | 1.225 |
|  |  | Mental | 1.328 | 0.039 | 1.249 | 1.407 |
|  |  | Music | 1.31 | 0.04 | 1.229 | 1.391 |
| Stride Length | Off | Uncued | 1.178 | 0.034 | 1.11 | 1.247 |
|  |  | Mental | 1.226 | 0.034 | 1.156 | 1.295 |
|  |  | Music | 1.174 | 0.038 | 1.096 | 1.251 |
|  | On | Uncued | 1.217 | 0.032 | 1.152 | 1.282 |
|  |  | Mental | 1.277 | 0.035 | 1.205 | 1.348 |
|  |  | Music | 1.238 | 0.036 | 1.164 | 1.312 |
| Double Limb Support % | Off | Uncued | 20.462 | 0.66 | 19.122 | 21.801 |
|  |  | Mental | 18.834 | 0.7 | 17.413 | 20.255 |
|  |  | Music | 19.365 | 0.716 | 17.911 | 20.819 |
|  | On | Uncued | 20.227 | 0.634 | 18.94 | 21.513 |
|  |  | Mental | 18.49 | 0.691 | 17.086 | 19.894 |
|  |  | Music | 18.667 | 0.705 | 17.236 | 20.097 |

**Pairwise Comparison Medication * Cue Condition A**

| Measure | Cue | Mean Difference | Std. Error | Sig.^b^ | 95% Confidence Interval for Difference^b^ | |
| --- | --- | --- | --- | --- | --- | --- |
|  |  |  |  |  | Lower Bound | Upper Bound |
| Cadence | Uncued | -0.454 | 0.704 | 0.523 | -1.883 | 0.975 |
|  | Mental | -0.593 | 0.688 | 0.394 | -1.99 | 0.803 |
|  | Music | 0.23 | 0.958 | 0.811 | -1.714 | 2.174 |
| Speed | Uncued | -.039* | 0.013 | 0.005 | -0.065 | -0.012 |
|  | Mental | -.056* | 0.013 | <.001 | -0.082 | -0.031 |
|  | Music | -.064* | 0.016 | <.001 | -0.097 | -0.031 |
| StrideLen | Uncued | -.039* | 0.011 | 0.001 | -0.061 | -0.016 |
|  | Mental | -.051* | 0.011 | <.001 | -0.073 | -0.029 |
|  | Music | -.064* | 0.013 | <.001 | -0.092 | -0.037 |
| DLS | Uncued | 0.235 | 0.152 | 0.13 | -0.073 | 0.543 |
|  | Mental | 0.344 | 0.171 | 0.053 | -0.004 | 0.692 |
|  | Music | .698* | 0.236 | 0.006 | 0.219 | 1.178 |
| Based on estimated marginal means | | | | | | |
| * The mean difference is significant at the .05 level. | | | | | | |
| b Adjustment for multiple comparisons: Bonferroni. | | | | | | |

**Pairwise Comparison Medication * Cue Condition B**

| Measure | Medication | (I) Cue | (J) Cue | Mean Difference (I-J) | Std. Error | Sig.^b^ | 95% Confidence Interval for Difference^b^ | |
| --- | --- | --- | --- | --- | --- | --- | --- | --- |
|  |  |  |  |  |  |  | Lower Bound | Upper Bound |
| Cadence | Off | Uncued | Mental | -10.885* | 0.913 | <.001 | -13.181 | -8.588 |
|  |  |  | Music | -14.203* | 1.297 | <.001 | -17.465 | -10.942 |
|  |  | Mental | Uncued | 10.885* | 0.913 | <.001 | 8.588 | 13.181 |
|  |  |  | Music | -3.318* | 0.728 | <.001 | -5.149 | -1.488 |
|  |  | Music | Uncued | 14.203* | 1.297 | <.001 | 10.942 | 17.465 |
|  |  |  | Mental | 3.318* | 0.728 | <.001 | 1.488 | 5.149 |
|  | On | Uncued | Mental | -11.024* | 1.057 | <.001 | -13.683 | -8.366 |
|  |  |  | Music | -13.519* | 1.389 | <.001 | -17.011 | -10.028 |
|  |  | Mental | Uncued | 11.024* | 1.057 | <.001 | 8.366 | 13.683 |
|  |  |  | Music | -2.495* | 0.716 | 0.004 | -4.295 | -0.695 |
|  |  | Music | Uncued | 13.519* | 1.389 | <.001 | 10.028 | 17.011 |
| Velocity | Off | Uncued | Mental | -.155* | 0.016 | <.001 | -0.196 | -0.113 |
|  |  |  | Music | -.129* | 0.021 | <.001 | -0.181 | -0.076 |
|  |  | Mental | Uncued | .155* | 0.016 | <.001 | 0.113 | 0.196 |
|  |  |  | Music | 0.026 | 0.011 | 0.066 | -0.001 | 0.053 |
|  |  | Music | Uncued | .129* | 0.021 | <.001 | 0.076 | 0.181 |
|  |  |  | Mental | -0.026 | 0.011 | 0.066 | -0.053 | 0.001 |
|  | On | Uncued | Mental | -.172* | 0.018 | <.001 | -0.217 | -0.127 |
|  |  |  | Music | -.154* | 0.018 | <.001 | -0.198 | -0.11 |
|  |  | Mental | Uncued | .172* | 0.018 | <.001 | 0.127 | 0.217 |
|  |  |  | Music | .018* | 0.006 | 0.021 | 0.002 | 0.034 |
|  |  | Music | Uncued | .154* | 0.018 | <.001 | 0.11 | 0.198 |
|  |  |  | Mental | -.018* | 0.006 | 0.021 | -0.034 | -0.002 |
| Stride Length | Off | Uncued | Mental | -.047* | 0.015 | 0.007 | -0.084 | -0.011 |
|  |  |  | Music | 0.004 | 0.02 | 1 | -0.047 | 0.055 |
|  |  | Mental | Uncued | .047* | 0.015 | 0.007 | 0.011 | 0.084 |
|  |  |  | Music | .052* | 0.011 | <.001 | 0.024 | 0.08 |
|  |  | Music | Uncued | -0.004 | 0.02 | 1 | -0.055 | 0.047 |
|  |  |  | Mental | -.052* | 0.011 | <.001 | -0.08 | -0.024 |
|  | On | Uncued | Mental | -.060* | 0.013 | <.001 | -0.092 | -0.028 |
|  |  |  | Music | -0.021 | 0.014 | 0.386 | -0.056 | 0.013 |
|  |  | Mental | Uncued | .060* | 0.013 | <.001 | 0.028 | 0.092 |
|  |  |  | Music | .039* | 0.007 | <.001 | 0.022 | 0.056 |
|  |  | Music | Uncued | 0.021 | 0.014 | 0.386 | -0.013 | 0.056 |
|  |  |  | Mental | -.039* | 0.007 | <.001 | -0.056 | -0.022 |
| Double Limb Support % | Off | Uncued | Mental | 1.628* | 0.193 | <.001 | 1.144 | 2.113 |
|  |  |  | Music | 1.097* | 0.21 | <.001 | 0.57 | 1.624 |
|  |  | Mental | Uncued | -1.628* | 0.193 | <.001 | -2.113 | -1.144 |
|  |  |  | Music | -.531* | 0.174 | 0.013 | -0.968 | -0.094 |
|  |  | Music | Uncued | -1.097* | 0.21 | <.001 | -1.624 | -0.57 |
|  |  |  | Mental | .531* | 0.174 | 0.013 | 0.094 | 0.968 |
|  | On | Uncued | Mental | 1.737* | 0.222 | <.001 | 1.179 | 2.294 |
|  |  |  | Music | 1.560* | 0.222 | <.001 | 1.002 | 2.118 |
|  |  | Mental | Uncued | -1.737* | 0.222 | <.001 | -2.294 | -1.179 |
|  |  |  | Music | -0.177 | 0.116 | 0.408 | -0.468 | 0.114 |
|  |  | Music | Uncued | -1.560* | 0.222 | <.001 | -2.118 | -1.002 |
|  |  |  | Mental | 0.177 | 0.116 | 0.408 | -0.114 | 0.468 |
| Based on estimated marginal means | | | | | | | | |
| * The mean difference is significant at the .05 level. | | | | | | | | |
| b Adjustment for multiple comparisons: Bonferroni. | | | | | | | | |

**Supplementary Data B: Estimated Marginal Means For MANOVA 2 (Variability Values of Cadence** **CV, Velocity CV, Stride Length CV, Double Limb Support % CV)**

**Grand Means**

| Measure | Mean | Std. Error | 95% Confidence Interval | |
| --- | --- | --- | --- | --- |
|  |  |  | Lower Bound | Upper Bound |
| Cadence CV | 0.341 | 0.017 | 0.307 | 0.376 |
| Velocity CV | 0.548 | 0.02 | 0.507 | 0.589 |
| Stride Length CV | 0.47 | 0.023 | 0.422 | 0.518 |
| Double Limb Support CV | 0.78 | 0.019 | 0.742 | 0.818 |

**Effect of Medication**

| Measure | Medication | Mean | Std. Error | 95% Confidence Interval | |
| --- | --- | --- | --- | --- | --- |
|  |  |  |  | Lower Bound | Upper Bound |
| Cadence CV | Off | 0.341 | 0.018 | 0.305 | 0.378 |
|  | On | 0.341 | 0.019 | 0.304 | 0.379 |
| Velocity CV | Off | 0.556 | 0.021 | 0.513 | 0.599 |
|  | On | 0.54 | 0.021 | 0.496 | 0.583 |
| Stride Length CV | Off | 0.484 | 0.025 | 0.434 | 0.534 |
|  | On | 0.456 | 0.025 | 0.406 | 0.506 |
| Double Limb Support CV | Off | 0.764 | 0.017 | 0.729 | 0.799 |
|  | On | 0.795 | 0.022 | 0.749 | 0.841 |

**Pairwise Comparison of Medication**

| Measure | Mean Difference | Std. Error | Sig.^b^ | 95% Confidence Interval for Difference^b^ | |
| --- | --- | --- | --- | --- | --- |
|  |  |  |  | Lower Bound | Upper Bound |
| Cadence CV | 0 | 0.014 | 0.99 | -0.028 | 0.028 |
| Velocity CV | 0.017 | 0.014 | 0.239 | -0.012 | 0.045 |
| Stride Length CV | 0.028 | 0.015 | 0.068 | -0.002 | 0.058 |
| Double Limb Support CV | -.031* | 0.015 | 0.044 | -0.061 | -0.001 |
| Based on estimated marginal means | | | | | |
| * The mean difference is significant at the .05 level. | | | | | |
| ^b^ Adjustment for multiple comparisons: Bonferroni. | | | | | |

**Effect of Cue**

| Measure | Cue | Mean | Std. Error | 95% Confidence Interval | |
| --- | --- | --- | --- | --- | --- |
|  |  |  |  | Lower Bound | Upper Bound |
| Cadence CV | Uncued | 0.295 | 0.021 | 0.253 | 0.337 |
|  | Mental | 0.309 | 0.019 | 0.27 | 0.349 |
|  | Music | 0.42 | 0.027 | 0.365 | 0.475 |
| Velocity CV | Uncued | 0.549 | 0.023 | 0.503 | 0.595 |
|  | Mental | 0.518 | 0.021 | 0.475 | 0.561 |
|  | Music | 0.576 | 0.023 | 0.53 | 0.623 |
| Stride Length CV | Uncued | 0.419 | 0.025 | 0.369 | 0.469 |
|  | Mental | 0.424 | 0.026 | 0.372 | 0.477 |
|  | Music | 0.567 | 0.03 | 0.506 | 0.628 |
| Double Limb Support CV | Uncued | 0.746 | 0.019 | 0.708 | 0.784 |
|  | Mental | 0.78 | 0.023 | 0.733 | 0.826 |
|  | Music | 0.814 | 0.022 | 0.77 | 0.857 |

**Pairwise Comparison of Cue**

| Measure | (I) Cue | (J) Cue | Mean Difference (I-J) | Std. Error | Sig.^b^ | 95% Confidence Interval for Difference^b^ | |
| --- | --- | --- | --- | --- | --- | --- | --- |
|  |  |  |  |  |  | Lower Bound | Upper Bound |
| Cadence CV | Uncued | Mental | -0.014 | 0.022 | 1 | -0.07 | 0.041 |
|  |  | Music | -.126* | 0.032 | 0.001 | -0.207 | -0.044 |
|  | Mental | Uncued | 0.014 | 0.022 | 1 | -0.041 | 0.07 |
|  |  | Music | -.111* | 0.022 | <.001 | -0.168 | -0.055 |
|  | Music | Uncued | .126* | 0.032 | 0.001 | 0.044 | 0.207 |
|  |  | Mental | .111* | 0.022 | <.001 | 0.055 | 0.168 |
| Velocity CV | Uncued | Mental | 0.031 | 0.016 | 0.201 | -0.01 | 0.072 |
|  |  | Music | -0.027 | 0.02 | 0.557 | -0.077 | 0.023 |
|  | Mental | Uncued | -0.031 | 0.016 | 0.201 | -0.072 | 0.01 |
|  |  | Music | -.058* | 0.013 | <.001 | -0.092 | -0.024 |
|  | Music | Uncued | 0.027 | 0.02 | 0.557 | -0.023 | 0.077 |
|  |  | Mental | .058* | 0.013 | <.001 | 0.024 | 0.092 |
| Stride Length CV | Uncued | Mental | -0.005 | 0.02 | 1 | -0.055 | 0.045 |
|  |  | Music | -.148* | 0.029 | <.001 | -0.221 | -0.074 |
|  | Mental | Uncued | 0.005 | 0.02 | 1 | -0.045 | 0.055 |
|  |  | Music | -.143* | 0.019 | <.001 | -0.19 | -0.096 |
|  | Music | Uncued | .148* | 0.029 | <.001 | 0.074 | 0.221 |
|  |  | Mental | .143* | 0.019 | <.001 | 0.096 | 0.19 |
| Double Limb Support CV | Uncued | Mental | -0.034 | 0.018 | 0.192 | -0.079 | 0.011 |
|  |  | Music | -.068* | 0.021 | 0.008 | -0.121 | -0.015 |
|  | Mental | Uncued | 0.034 | 0.018 | 0.192 | -0.011 | 0.079 |
|  |  | Music | -.034* | 0.011 | 0.011 | -0.061 | -0.007 |
|  | Music | Uncued | .068* | 0.021 | 0.008 | 0.015 | 0.121 |
|  |  | Mental | .034* | 0.011 | 0.011 | 0.007 | 0.061 |
| Based on estimated marginal means | | | | | | | |
| * The mean difference is significant at the .05 level. | | | | | | | |
| ^b^ Adjustment for multiple comparisons: Bonferroni. | | | | | | | |
